# Family-based childhood obesity prevention interventions: a systematic review and quantitative content analysis

**DOI:** 10.1186/s12966-017-0571-2

**Published:** 2017-08-24

**Authors:** Tayla Ash, Alen Agaronov, Ta’Loria Young, Alyssa Aftosmes-Tobio, Kirsten K. Davison

**Affiliations:** 1 Harvard T.H. Chan School of Public Health, Department of Social and Behavioral Sciences, SPH-2 655 Huntington Avenue, Boston, 02115 USA; 2 Harvard T.H. Chan School of Public Health, Department of Nutrition, Kresge Building 677 Huntington Avenue, Boston, 02115 USA; 3 Harvard T.H. Chan School of Public Health, University of Texas at Austin, 110 Inner Campus Drive, Austin, 78705 USA

**Keywords:** Childhood obesity, Diet, Physical activity, Media use, Sedentary behavior, Sleep, Family-based

## Abstract

**Background:**

A wide range of interventions has been implemented and tested to prevent obesity in children. Given parents’ influence and control over children’s energy-balance behaviors, including diet, physical activity, media use, and sleep, family interventions are a key strategy in this effort. The objective of this study was to profile the field of recent family-based childhood obesity prevention interventions by employing systematic review and quantitative content analysis methods to identify gaps in the knowledge base.

**Methods:**

Using a comprehensive search strategy, we searched the PubMed, PsycIFO, and CINAHL databases to identify eligible interventions aimed at preventing childhood obesity with an active family component published between 2008 and 2015. Characteristics of study design, behavioral domains targeted, and sample demographics were extracted from eligible articles using a comprehensive codebook.

**Results:**

More than 90% of the 119 eligible interventions were based in the United States, Europe, or Australia. Most interventions targeted children 2–5 years of age (43%) or 6–10 years of age (35%), with few studies targeting the prenatal period (8%) or children 14–17 years of age (7%). The home (28%), primary health care (27%), and community (33%) were the most common intervention settings. Diet (90%) and physical activity (82%) were more frequently targeted in interventions than media use (55%) and sleep (20%). Only 16% of interventions targeted all four behavioral domains. In addition to studies in developing countries, racial minorities and non-traditional families were also underrepresented. Hispanic/Latino and families of low socioeconomic status were highly represented.

**Conclusions:**

The limited number of interventions targeting diverse populations and obesity risk behaviors beyond diet and physical activity inhibit the development of comprehensive, tailored interventions. To ensure a broad evidence base, more interventions implemented in developing countries and targeting racial minorities, children at both ends of the age spectrum, and media and sleep behaviors would be beneficial. This study can help inform future decision-making around the design and funding of family-based interventions to prevent childhood obesity.

**Electronic supplementary material:**

The online version of this article (doi:10.1186/s12966-017-0571-2) contains supplementary material, which is available to authorized users.

## Background

Childhood obesity continues to be a pervasive global public health issue as children worldwide are significantly heavier than prior generations [1]. Over the past few decades, the prevalence of obesity among children and adolescents has risen by 47% [2]. Increases have been seen in both developed and developing countries, with recent prevalence estimates of 23 and 13%, respectively [2]. Despite evidence of a plateau in the rates of obesity, at least among young children in developed countries, current levels are still too high, posing short- and long-term impacts on children’s physical, psychological, social, and economic well-being [2–5]. Of equal, if not greater concern, racial/ethnic and socioeconomic disparities appear to be widening in some countries [5–8]. Given the extensive disease burden, treatment resistance of obesity, and lack of signs of attenuation for rates in the developing world, scientists, clinicians, and practitioners are working hard to devise and test interventions to prevent childhood obesity and reduce associated disparities [2, 9].

One category of interventions to prevent childhood obesity that has grown considerably in recent years is family-based interventions. This was in part due to a number of key reports published in 2007, including an Institute of Medicine (IOM) report on the recent progress of childhood obesity prevention [10] and a report from a committee of experts representing 15 professional organizations appointed to make evidence-based recommendations for the prevention, assessment, and treatment of childhood obesity [11, 12]. In both reports, parents are described as integral targets in interventions, given their highly influential role in supporting and managing the four behaviors that affect children’s energy balance (diet, physical activity, media use, and sleep) [13–15]. This includes not only parenting practices and rules, but also the environments to which children are exposed, and the adoption of parents’ own behavioral habits by children [15–19].

Since the release of these reports, there has been a proliferation of family-based interventions to prevent and treat childhood obesity as documented in at least five published reviews of this literature in the past decade [20–24]. While these reviews convey extensive information around intervention effectiveness, they cannot reveal gaps in the knowledge base. Quantitative content analysis [25–27] can be used to code intervention and participant characteristics, and a review of the resulting data can reveal areas and populations receiving a great deal of attention, as well as those where few or no studies exist, thereby highlighting knowledge gaps. With a focus on childhood obesity interventions, pertinent questions to address include: whether interventions have continued to focus primarily on diet and physical activity, neglecting the more recently established predictors of media use and sleep [28–30]; whether some behaviors are more likely to be targeted among certain age groups or settings than others; and whether there are gaps with regard to the populations targeted by interventions to date, in particular, the representation of vulnerable populations (e.g. families living in developing countries, those of low socioeconomic status, racial and ethnic minorities, immigrants, and non-traditional families) [2, 31–37]. In addition to ethical reasons, from a pragmatic viewpoint, it is difficult to identify best practices to prevent childhood obesity in vulnerable populations when few interventions have focused on that population [38, 39].

The goal of this study is to profile family-based interventions to prevent childhood obesity published since 2008 to identify gaps in intervention design and methodology. In particular, we use quantitative content analysis to systematically document intervention and sample characteristics with the goal of directing future research to address the identified knowledge gaps.

## Methods

We used a multistage process informed by the Preferred Reporting Items for Systematic Reviews and Meta-Analyses (PRISMA) guidelines to identify family-based childhood obesity prevention interventions that were written in English and published between January 1, 2008 and December 31, 2015 [40]. Using an a priori defined protocol, we identified relevant articles and systematically screened articles against inclusion and exclusion criteria. The systematic review protocol was registered in the PROSPERO database (CRD42016042009).

Following the identification of eligible studies, we conducted a quantitative content analysis to profile recent interventions for childhood obesity prevention. Content analysis, originally used in communication sciences but increasingly utilized in public health, is a research method used to generate objective, systematic, and quantitative descriptions of a topic of interest [25–27]. Our research team has previously employed this technique to survey observational studies on parenting and childhood obesity published between 2009 and 2015 [41, 42].

### Search strategy and initial screening

With the help of a research librarian, two authors (TA, AA) searched three databases (PubMed, PsycINFO, and CINAHL) using individually tailored search strategies most appropriate for each database. The selected databases are the three most common databases used in recent systematic reviews. Our search strategy consisted of search strings composed of terms targeting four concepts: (1) family (e.g. family, mother, father, home), (2) intervention (e.g. prevention, promotion), (3) children (e.g. child, infant, youth), and (4) obesity (e.g. overweight, body mass) (see Additional file 1 for full search strategy for one database). We searched title, abstract, and medical subject headings (MeSH) or descriptor subjects (DE) term fields. Animal studies (e.g. rats), non-original research articles (e.g. commentaries, editorials, case reports), studies written in languages other than English and studies focused on populations older than 18 years were excluded using search limits and NOT terms. We restricted the search to articles published since January 1, 2008, to capture interventions implemented after the release of the IOM and expert committee reports. Furthermore, a start point of January 2008 ensured the feasibility of this study given the labor and time intensive process to screen and code studies. In a recent systematic review of family-based interventions for the treatment and prevention of childhood obesity, more than 80% of eligible studies were published since 2008 [43]. Thus, a start date of 2008 appropriately balances feasibility of implementation and the validity of the resulting information. The search end date was December 31, 2015.

**Fig. 1:**
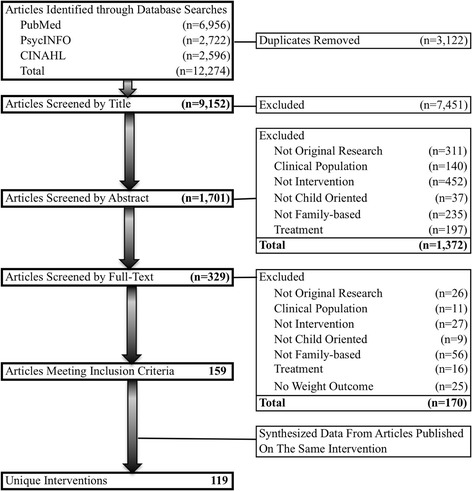
PRISMA flow diagram for identifying and screening eligible family-based childhood obesity prevention interventions

The search yielded 12,274 hits, representing 9152 unique articles after removing duplicates (see Fig. 1). Following a review of titles by three authors (TA, AA, TY) and one research assistant, 7451 articles were removed based on exclusion criteria, resulting in 1701 articles that proceeded to abstract review. Articles were removed during title review if they were not written in English or published in the designated time frame, were not original research articles, did not include human subjects, did not target children, were observational studies, were not relevant to the topic of childhood obesity (e.g. papers about Anorexia Nervosa), or included special clinical populations.

### Application of eligibility criteria

Three authors (TA, AA, TY) and one research assistant screened articles against the eligibility criteria during abstract review, while two authors (TA, AA) screened during full-text review, applying the aforementioned exclusion criteria. Eligible studies included family-based interventions for childhood obesity prevention published since 2008. We defined *family-based* interventions as those involving active and repeated involvement in intervention activities from at least one parent or guardian [19]. Examples of intervention activities that qualify as active parent involvement include workshops and counseling. Examples of passive involvement, which were excluded, include sending home brochures for parents, or simply inviting parents to a single event, but not involving them in the intervention in an integral way. We defined *obesity* interventions as those that reported at least one weight-related outcome (weight, body mass index, etc.) or which self-identified as an obesity intervention. We defined interventions as *preventive* if they did not explicitly focus on weight loss or management, or if they did not recruit only children with obesity. The final inclusion criterion was that the intervention was designed with the intent of benefiting children (child being defined as <18 years of age), excluded interventions in which the objective was to better parent health outcomes.

Of the 1701 articles screened at the abstract level, 329 proceeded to full-text screening, of which 159 articles met the eligibility criteria and were included in the final pool of eligible papers (see Additional file 2 for a list of eligible articles). We examined intervention name, trial number, the last name of the first author, and the last name of the last author to identify articles that originated from the same intervention. After collating, 119 unique interventions were identified, which included interventions with published outcome data, and interventions for which only a protocol was published. Percent agreement for all screening criteria ranged between 86 and 98%. Discrepancies were discussed and resolved.

To ensure a fully inclusive search strategy, we also reviewed the references of a random subset of the articles meeting the inclusion criteria. A subset of 5% was chosen given the large sample size. No additional studies meeting the eligibility criteria were identified in the process, suggesting that the employed search was exhaustive.

### Data extraction

For all eligible articles, we used conventional content analysis methodology [25–27] to extract and analyze article, intervention, and participant characteristics. We developed a comprehensive codebook to standardize the coding process. Multiple authors (TA, AA, AA-T) tested the codebook by coding five articles not included in the final pool of studies. An additional round of testing included 10 randomly selected articles from the study pool. After pilot testing the codebook and establishing reliability (see intercoder reliability), two trained coders (TA, AA) each coded half of the 159 eligible articles.

#### Article characteristics

We coded publication year, journal, funding sources, and type of paper. All specific funding sources for a given intervention were extracted and classified after web-based searching. Funding sources were categorized as federal, foundation, corporate, or university, and then further coded based on the specific federal, foundation or corporate agency. For type of paper, articles were coded as an intervention protocol or outcome evaluation. Articles that reported any intervention outcomes were coded as outcome evaluations; interventions that only described the intervention (or provided only baseline data) were coded as protocols. Because a seemingly large number of protocols were discovered among the final pool of articles, we elected to include them in the study. Interventions in which only a protocol has been published tend to represent the next generation of intervention studies and thus lend to a better understanding of the field’s trajectory.

#### Intervention characteristics

We coded a wide range of intervention characteristics including geographic region of the study, age of target child, intervention setting, length of intervention, delivery mode, evaluation design, intervention recipient, behavioral domains targeted, and theory used. Age of the target child at baseline was coded as prenatal (i.e., the intervention started before birth), 0–1 years, 2–5 years, 6–10 years, 11–13 years, and 14–17 years. If the age range fell predominantly into one category, any subsequent categories were only coded affirmative if the ages of participants crossed at least 2 years into a given range. Intervention setting was coded as home, primary care or health clinic, community-based, school, and childcare/preschool. Community-based interventions included those taking place in community gardens, parks, or recreational facilities. Interventions taking place at universities were also coded as community-based. In cases where intervention setting was ambiguous, or the intervention was not setting specific, we coded the intervention setting as unclear.

Intervention length was coded as less than 13 weeks (3 months), 13–51 weeks (3–11.9 months), or 52 weeks (12 months) or more. Two different types of intervention delivery modes were coded: in-person and technology-based. Technology-based approaches included those using computers, social media, text messages, or anything else involving the Internet. Evaluation design was coded as either randomized-controlled trial or quasi-experimental trial. We also extracted data on intervention recipients (i.e. those who directly received the intervention program or materials). This was coded as adults, children, or both. Behavioral domains targeted included diet, physical activity, media use, and sleep. Finally, we coded use of theory. Theories were specified using the following categories: social cognitive theory, parenting styles, ecological frameworks, transtheoretical model of behavior change, health belief model, theory of planned behavior, or other. For age category, intervention setting, delivery mode, intervention recipients, and theory, multiple categories could be selected.

#### Sample characteristics

Sample characteristics were coded for the inclusion of participants from underserved populations and non-traditional families, and racial/ethnic composition of the sample. We coded sample characteristics for outcome evaluations only (*n* = 84 studies) because intervention protocols generally do not include this information. We coded whether the intervention included any participants from the following underserved or non-traditional groups: low socioeconomic status (SES), racial/ethnic minorities (i.e., Black/African American, Hispanic/Latino, Indigenous), immigrant families, single parents, non-biological parents, and non-residential parents. Low SES was defined as either low income (self-identified by the study) or low education (high school diploma or less). Families participating in low-income qualifying programs (Women, Infants, and Children services, Supplemental Nutrition Assistance Program, free or reduced school lunch, Head Start, etc.) were considered low SES. We coded parents as single if they self-identified as such, were not cohabitating, or were widowed or divorced. In studies where limited information was provided and marital status was simply dichotomized as married or not married, not married was used as a proxy for single. Finally, we coded whether the sample included participants from each racial/ethnic group (i.e. White, Black/African American, Hispanic/Latino, Asian, Indigenous, and multiracial/other). For all sample characteristics, in addition to coding whether families belonging to each of the groups were included, we also coded whether they made up at least 50% of the sample, as well as 90% of the sample. The purpose of these categories was to distinguish between studies that included only a few families from a given category and those in which at least half the sample belonged to the category. If at least 90% of the families included in a sample belonged to a given category, the sample was considered to be predominantly that category (e.g. predominantly-Hispanic). Samples coded affirmative for 90% criteria were also coded affirmative for the 50% criteria.

### Inter-rater reliability

Both coders coded randomly selected articles from the final study pool until reliability was sufficiently established. Ultimately, this included four rounds of coding a total of 55 articles. We computed Cohen’s kappa as a measure of agreement between the coders, using weighted kappas for ordinal variables [44]. The final average kappa across all variables was 0.87, and the average percent agreement was 92%. Three variables had kappas below 0.70, the conservative threshold for adequate inter-rater reliability [45]. These variables included the following: inclusion of children 11–13 years old (kappa 0.36), inclusion of children 14–17 years old (kappa 0.65), and childcare/preschool setting (kappa 0.46). Because percent agreement for each of these variables was high (>89%), and given that kappa coefficients are difficult to interpret when variability is low [45, 46], which would result from a category (e.g. inclusion of children 14–17 years) being infrequently coded or endorsed, they were retained in the analyses. Coders were retrained on the three variables prior to coding the remainder of the articles.

### Data synthesis and analysis

Both inter-rater reliability and all other analyses were conducted in STATA 13 [StataCorp LP, College Station, TX, USA]. One coder (TA) cleaned the data. The majority of missing data was not reported (i.e., were missing by design) and therefore coded as ‘0’ (no/not sure). Where data were missing, one of the coders (TA) returned to the full-text article to confirm and correct any errors.

For article characteristics (e.g. publication year, journal), the unit of analysis is article, with a denominator of 159 articles. For intervention and sample characteristics, which are presented in Tables 1-3, the unit of analysis is intervention. In instances where multiple studies were published on the same intervention, the data extracted from each study were synthesized into a single entry [47]. For example, if both a protocol and outcome evaluation were published for an intervention, the intervention was marked as having an outcome evaluation. As a result, a denominator of 119 interventions was used to assess intervention characteristics. Interventions with a protocol only were not included in the assessment of sample characteristics because sample information is infrequently reported in such papers. Thus the denominator for sample characteristics was 85 interventions with published outcome data.

**Table 1:** Intervention characteristics of family-based childhood obesity prevention interventions published from 2008 to 2015 (*n* = 119)

	n (%)
Geographic Region	
Unites States	66 (56)
Europe/United Kingdom	30 (25)
Australia/New Zealand	10 (8)
Canada	6 (5)
Other^a^	7 (6)
Age of target child^b^	
Prenatal	10 (8)
0–1 years (toddler)	29 (24)
2–5 years (preschool-kindergarten)	51 (43)
6–10 years (elementary school)	42 (35)
11–13 years (middle school)	25 (21)
14–17 years (high school)	8 (7)
Setting^b^	
Home	33 (28)
Primary care/health clinic	32 (27)
Community-based	39 (33)
School	21 (18)
Childcare/preschool	11 (9)
Multi-setting	24 (20)
Not setting specific/Unclear	11 (9)
Length of intervention	
< 13 weeks (<3 months)	35 (29)
13–51 week (3–11.9 months)	47 (40)
52 weeks or more (12 months or more)	33 (28)
Unclear	4 (3)
Delivery approach^b^	
In-person delivery	101 (85)
Technology-based delivery	27 (23)
Evaluation Design	
Randomized-controlled trial design	87 (73)
Recipients of intervention activities^b^	
Children	65 (55)
Adults	119 (100)
Behavioral domains targeted^b^	
Diet	107 (90)
Physical activity	97 (82)
Media use	65 (55)
Sleep	24 (20)
Funding source^b^	
Federal	75 (63)
Foundation	50 (42)
Corporate	21 (18)
University	23 (19)
Unclear	8 (7)
Type of paper	
Outcome evaluation	85 (71)
Protocol only	34 (29)
Theory^b^	
Social Cognitive Theory	49 (41)
Parenting Styles	20 (17)
Ecological Framework	20 (17)
Transtheoretical Model of Behavior Change	10 (8)
Health Belief Model	8 (7)
Theory of Planned Behavior	6 (5)
Other	23 (19)
Unclear	34 (29)

^a^Other: Mexico/Central America- 2, South America- 2, Asia- 2, Middle East- 1; ^b^Groups are not mutually exclusive thus totals may exceed 100%

We also examined article and intervention characteristics separately for protocols and outcome evaluations. Given that few differences were identified, this information is presented in Additional file 3: Table S1 to streamline the presentation of results.

## Results

The number of eligible articles published each year was as follows: 2008 = 6 (4%), 2009 = 5 (3%), 2010 = 14 (9%), 2011 = 15 (9%), 2012 = 33 (21%), 2013 = 35 (22%), 2014 = 23 (14%), and 2015 = 28 (18%). The predominant journals in which articles were published included BioMed Central Public Health (*n* = 28, 18%), Contemporary Clinical Trials (*n* = 12, 8%), Childhood Obesity (*n* = 9, 6%), Pediatrics (*n* = 7, 4%), Pediatric Obesity (*n* = 6, 4%), and Preventive Medicine (*n* = 6, 4%).

### Intervention characteristics

Eligible articles described 119 unique interventions. Table 1 summarizes additional intervention characteristics for eligible interventions. For more than a fourth of these interventions (*n* = 34, 29%), only an intervention protocol was identified (i.e., no published outcomes were available). More than half (*n* = 66, 56%) of the interventions were based in the U.S. Studies based in Europe/United Kingdom (*n* = 30, 25%), Australia/New Zealand (*n* = 10, 8%), and Canada (*n* = 6, 5%) comprised 38%. Few interventions were conducted in countries in Central America, South America, Asia, Africa, the Middle East, or the Caribbean.

Less than a third of interventions were implemented for a year or more (*n* = 33, 28%). Interventions that were implemented in-person (*n* = 101, 85%) were more common than those delivered using technology (*n* = 27, 23%). Fourteen (12%) of interventions had both in-person and technology components. Five interventions (4%) had neither an in-person nor a technology component; these interventions consisted of printed materials and phone calls. Nearly three out of four interventions utilized a randomized controlled trial design (*n* = 87, 73%). Because active parent engagement was a requirement for eligibility in this review, parents were intervention recipients in all interventions. Children were also intervention recipients in approximately half of the interventions (*n* = 65, 55%).

A slight majority of interventions were federally funded (*n* = 75, 63%). Of these, about half (*n* = 34, 29% of the 119 eligible interventions) received funding from the National Institutes of Health, with the National Institute of Diabetes and Digestive and Kidney Diseases (*n* = 14, 12%) and the National Heart, Lung, and Blood Institute (*n* = 7, 6%) being the two leading funding institutes (data not shown). The United States Department of Agriculture funded 10 (8%) interventions. Twenty-three (19%) interventions received federal funding from countries other than the United States, with Australia funding the most (*n* = 6, 5%). Of the 50 (42%) interventions funded by foundations, the Robert Woods Johnson Foundation was the leading funder (*n* = 5, 4%). A similar proportion of interventions received corporate (*n* = 21, 18%) or university funding (*n* = 23, 19%). Many interventions (*n* = 46, 39%) received multiple types of funding, and funding source was not listed in 8 (7%) of interventions.

A majority of interventions mentioned theory (*n* = 85, 71%), with many (*n* = 34, 29%) using multiple theories. However, interventions varied greatly with respect to how heavily theory was emphasized. Social cognitive theory was the most widely noted theory (*n* = 49, 41%).

Approximately 40% of interventions targeted families with children ages 2–5 years (*n* = 51, 43%) or 6–10 years (*n* = 42, 35%), whereas fewer than 10% of interventions targeted families during the prenatal period (*n* = 10, 8%) or families of children with 14–17-year-olds (*n* = 8, 7%). One in three interventions were implemented in a home setting (*n* = 33, 28%), a primary care/health clinic (*n* = 32, 27%) or in the community (*n* = 39, 33%), and one in five (*n* = 24) were implemented in multiple settings. Finally, just over half (*n* = 69, 58%) of studies targeted a behavioral domain beyond diet and physical activity (i.e., they targeted media use and/or sleep in addition to diet and physical activity), and only a few (*n* = 3, 3%) interventions did not target either diet or physical activity.

Table 2 provides a cross tabulation of age of target child, setting, and behavioral domains. A number of patterns are apparent. First, interventions that targeted children in the earlier years of life (prenatal to age 5 years) tended to be focused in the home (*n* = 28, 31%) and primary care settings (*n* = 30, 33%), whereas interventions that targeted older children occurred most frequently in community (*n* = 40, 53%) and school (*n* = 20, 27%) settings. Second, media use was least frequently included in school-based interventions (*n* = 9, 43%). Physical activity was most frequently targeted in a school setting (*n* = 21, 100%), and least likely to be targeted in homes (*n* = 23, 70%). Sleep was most often included in home-based (*n* = 8, 24%), health-based (*n* = 8, 25%), and childcare-based (*n* = 3, 27%) interventions; it was seldom targeted in families with school-age children (*n* = 4, 10%) and has not been targeted in families with children older than 10 years of age.

**Table 2:** Age of target child, setting, and behavioral domains targeted of family-based childhood obesity prevention interventions published 2008–2015 (*n* = 119)

			Age of target child^a^						
Setting^a^	n (%)	Bx Dom^a^	All ages	Prenatal	0–1 years	2–5 years	6–10 years	11–13 years	14–17 years
	All settings		119 (100)	10 (8)	29 (24)	51 (43)	42 (35)	25 (21)	8 (7)
		D	107 (90)	10 (100)	27 (93)	47 (92)	38 (90)	23 (92)	7 (88)
		PA	97 (82)	8 (80)	17 (59)	42 (82)	37 (88)	22 (88)	7 (88)
		M	65 (55)	4 (40)	15 (52)	34 (67)	22 (52)	13 (52)	4 (50)
		S	24 (20)	4 (40)	9 (31)	14 (27)	4 (10)	0 (0)	0 (0)
	Home		33 (28)	4 (40)	11 (38)	13 (25)	8 (19)	6 (24)	1 (13)
		D	29 (88)	4 (100)	10 (91)	13 (100)	6 (75)	5 (83)	1 (100)
		PA	23 (70)	4 (100)	6 (55)	8 (62)	7 (88)	5 (83)	1 (100)
		M	20 (61)	4 (100)	7 (64)	9 (69)	3 (38)	3 (50)	1 (100)
		S	8 (24)	3 (75)	5 (45)	2 (15)	0 (0)	0 (0)	0 (0)
	Primary care/health clinic		32 (27)	4 (40)	14 (48)	12 (24)	8 (19)	5 (20)	2 (25)
		D	30 (94)	4 (100)	13 (93)	11 (92)	8 (100)	5 (100)	2 (100)
		PA	28 (88)	4 (100)	11 (79)	11 (92)	8 (100)	5 (100)	2 (100)
		M	19 (59)	1 (25)	7 (50)	10 (83)	6 (75)	3 (60)	1 (50)
		S	8 (25)	1 (25)	5 (36)	3 (25)	1 (13)	0 (0)	0 (0)
	Community-based		39 (33)	2 (20)	3 (10)	15 (29)	22 (52)	14 (56)	4 (50)
		D	36 (92)	2 (100)	3 (100)	15 (100)	19 (86	13 (93)	4 (100)
		PA	34 (87)	2 (100)	2 (67)	14 (93)	18 (82)	11 (79)	3 (75)
		M	25 (64)	1 (50)	2 (67)	11 (73)	13 (59)	8 (57)	2 (50)
		S	6 (15)	1 (50)	0 (0)	5 (33)	1 (5)	0 (0)	0 (0)
	School		21 (18)	0 (0)	0 (0)	8 (16)	13 (31)	5 (20)	2 (25)
		D	18 (86)	0 (0)	0 (0)	6 (75)	13 (100)	5 (100)	1 (50)
		PA	21 (100)	0 (0)	0 (0)	8 (100)	13 (100)	5 (100)	2 (100)
		M	9 (43)	0 (0)	0 (0)	4 (50)	6 (46)	2 (40)	1 (50)
		S	3 (14)	0 (0)	0 (0)	2 (25)	2 (15)	0 (0)	0 (0)
	Childcare/preschool		11 (9)	0 (0)	1 (3)	11 (22)	2 (5)	1 (4)	0 (0)
		D	9 (82)	0 (0)	1 (100)	9 (82)	2 (100)	1 (100)	0 (0)
		PA	11 (100)	0 (0)	1 (100)	11 (100)	2 (100)	1 (100)	0 (0)
		M	8 (73)	0 (0)	1 (100)	8 (73)	1 (50)	0 (0)	0 (0)
		S	3 (27)	0 (0)	0 (0)	3 (27)	1 (50)	0 (0)	0 (0)
	Multi-setting		24 (20)	2 (20)	3 (10)	12 (24)	9 (21)	6 (24)	2 (25)
		D	21 (88)	2 (100)	3 (100)	10 (83)	8 (89)	6 (100)	2 (100)
		PA	23 (96)	(100)	3 (100)	12 (100)	8 (89)	5 (83)	2 (100)
		M	19 (79)	2 (100)	3 (100)	9 (75)	6 (67)	5 (83)	2 (100)
		S	7 (29)	2 (100)	2 (67)	4 (33)	1 (11)	0 (0)	0 (0)
	Not setting specific/Unclear		11 (9)	1 (10)	2 (7)	7 (14)	1 (2)	2 (8)	1 (13)
		D	10 (91)	1 (100)	2 (100)	6 (86)	1 (100)	2 (100)	1 (100)
		PA	8 (73)	0 (0)	0 (0)	6 (86)	1 (100)	2 (100)	1 (100)
		M	6 (55)	0 (0)	1 (50)	3 (43)	1 (100)	2 (100)	1 (100)
		S	3 (27)	0 (0)	0 (0)	3 (43)	0 (0)	0 (0)	0 (0)

Setting, age, and domain groups are not mutually exclusive thus totals may exceed 100%

*Bx Dom* behavioral domain targeted, *D* diet, *PA* physical activity, *M* media use, *S* sleep

### Sample characteristics

Sample characteristics are summarized in Table 3. Underserved families appeared well-represented, particularly low SES families (*n* = 62, 73%). A slight majority of samples included at least some racial or ethnic minority families (*n* = 46, 54%), and just over a quarter included immigrant families (*n* = 24, 28%). Ethnic minorities (i.e., Hispanics) were better represented than racial minorities. About half of the interventions included families identifying as Hispanic/Latino (*n* = 40, 47%).

**Table 3:** Sample characteristics for family-based childhood obesity prevention interventions published from 2008 to 2015 (*n* = 85)^a^

	n (%)
Representation of underserved populations^b^	
Low SES (income or education)	62 (73)
Racial/ethnic minorities	46 (54)
Immigrants	24 (28)
Non-traditional families^b^	
Single parents	23 (27)
Non-biological parents	2 (2)
Non-residential parents	0 (0)
Racial/ethnic groups^b^	
White	30 (35)
Black/African American	26 (31)
Hispanic/Latino	40 (47)
Asian	20 (24)
Indigenous	12 (14)
Multiracial/Other	24 (28)
Unclear	29 (34)

^a^Sample characteristics are only provided for interventions with evaluations

^b^Groups are not mutually exclusive thus totals may exceed 100%

**Fig. 2:**
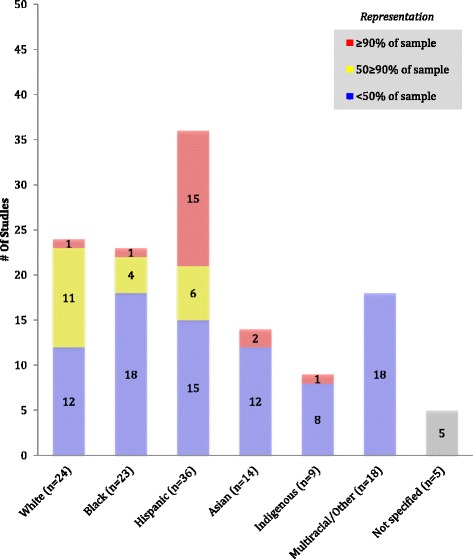
Inclusion and representation for racial/ethnic groups in U.S. family-based childhood obesity prevention interventions (*n* = 50)

The most frequently represented racial group was White (*n* = 30, 35%), followed by Black/African American (*n* = 26, 31%), Asian (*n* = 20, 24%), and then Indigenous (*n* = 12, 14%). Notably, many interventions (*n* = 29, 34%) did not specify the racial/ethnic background of families. Fig. 2 provides a more detailed assessment of the racial/ethnic composition of U.S.-based interventions (non-U.S. interventions infrequently reported participant race or ethnicity and were therefore not included). In 42% (*n* = 21) of U.S.-based interventions, Hispanic/Latino families made up at least half of the sample, and in 30% (*n* = 15) of interventions they made up at least 90% of the sample. Again, families identifying as White were the most represented racial group (*n* = 24, 48%). Less than 20% of studies included a sample that was at least half Black/African American (*n* = 5, 10%), Asian (*n* = 2, 4%), or Indigenous (*n* = 1, 2%).

Few studies included non-traditional families; less than a third of interventions included any single parent households (*n* = 23, 27%) and less than 5% included non-biological parents (*n* = 2, 2%) or non-residential parents (*n* = 0, 0%).

### Comparing protocols to outcome evaluations

When comparing interventions with evaluations to those with protocols only, a proxy for more recent interventions, interventions with protocols targeted more domains than those with evaluations. The proportion of evaluation and protocols that targeted just one behavioral domain was 20 and 12%, respectively, while the proportion targeting all four behavioral domains was 13 and 24%, respectively. Other notable differences were that interventions with protocols only were more likely to be of longer duration, utilize technology, adopt a randomized controlled trial design, target parents exclusively, receive federal funding, and use theory (see Additional file 3: Table S1).

## Discussion

Parents are important agents of change in the childhood obesity epidemic [20, 22, 48, 49]. This study used rigorous systematic methods to conduct a quantitative content analysis of family-based interventions to prevent childhood published between 2008 and 2015 to profile the field of recent family-based childhood obesity prevention interventions and identify knowledge gaps. We identified gaps in both intervention content and sample demographics. Key research gaps include studies in low-income countries, interventions for children on both the lower and higher ends of the age spectrum, and interventions targeting media use and sleep. Racial minorities and children from non-traditional families have also been underrepresented.

### Intervention gaps and implications

The vast majority of studies were conducted in developed, or high-income, countries. Given the rapid increase of obesity as a significant public health burden in developing countries, this study demonstrates a need for further intervention efforts in low- and middle-income countries [50, 51]. Although obesity rates are lower in low- and middle-income countries than developed countries, two-thirds of people with obesity worldwide live in developing countries where rates of obesity are increasing [2]. The small number of studies in these geographic regions limits the development of locally relevant programs and policies aiming to address the growing problem of obesity in these regions.

Non-traditional families were underrepresented in interventions. This is concerning given that children from non-traditional families have an elevated risk for obesity [31–36]. The changing nature of family structures, including the increasing number of single-parent households over time, [52] calls for a more inclusive approach to defining what is considered a family in research. Like non-traditional families, Black/African American, Asian, and Indigenous families have been underrepresented. Racial and ethnic minorities are vulnerable populations who experience elevated risk for obesity [33, 34]. Initiatives to fund interventions specifically targeted at racial and ethnic minorities may have increased the number of interventions targeting Hispanics, but not racial minorities. Thus, more efforts are needed that specifically target families identifying as races other than White. The lack of studies including adequate representation of these groups limits the scientific community’s understanding of effective strategies in high-risk communities and fails to fully address noted health disparities.

Family-based childhood obesity prevention interventions have focused heavily on children 2–10 years of age, despite the robust evidence demonstrating the importance of prevention efforts as early as infancy and the prenatal period [53, 54]. Establishing healthy habits early in life is critical given the difficulty of changing energy-balance behaviors later on. While it has been established that prenatal life influences childhood obesity risk, the low number of interventions beginning in the prenatal period, in particular, may be due to a general lack of understanding of the mechanisms responsible for this association, and general debate in the field about how early intervention efforts should begin [55, 56].

This study also revealed gaps in behavioral domains targeted, as interventions have not adequately targeted media use and sleep. Moreover, only 16% of interventions targeted all four behavioral domains. The emphasis of interventions on diet and physical activity may reflect their relative contribution to obesity risk. However, behavioral risk factors for obesity are interconnected, and thus may be better addressed by considering complimentary and supplementary behaviors [57–59]. While it can be argued that targeted messages may have a greater impact, the research gaps identified in this study (e.g. the lack of interventions targeting sleep among older children) highlight areas of needed research in the field. It is worth acknowledging how varied intervention length was across studies, with about a third of interventions being less than 3 months long. This is important given the difficulty in making and sustaining lifestyle changes.

### Comparisons with observational studies

The results of this study are consistent with findings from a content analysis by Gicevic et al. on observational research on parenting and childhood obesity published over a similar time frame [41]. The majority of studies were conducted in developed countries; diet and physical activity were the most heavily targeted behavioral domains; most studies targeted children ages 2–10; and there was a low representation, or at least specification, of non-traditional families. Also consistent with Gicevic et al., non-U.S. studies seldom reported the racial/ethnic composition of the sample [41].

### Limitations

There are several limitations to this study that are worth noting. First, this study focused on articles published over a relatively narrow time-period. Given the immense number of records initially identified, we needed to consider the feasibility of screening and then thoroughly coding eligible articles. Thus we decided to focus on recent literature. Additionally, it was not a focus of this study to look at time trends. Future studies that wish to see how the field is changing should do time-trend analyses, ideally taking into account a longer period of time. Another limitation of this study is that we did not assess intervention effectiveness or quality. While this may limit the potential utility of this review, we chose to focus on the results of the content analysis and not include this information because it is included in prior reviews of family-based interventions for childhood obesity prevention published in the past 10 years [20–24, 60]. Although systematic reviews can identify effective intervention strategies, they cannot identify the absence of information or gaps in the literature. This study explicitly addressed this shortfall in prior reviews. Lastly, the results of this study may be influenced by the number and choice of databases searched, and may be subject to publication bias. Given the large volume of studies (~7000) obtained by searching PubMed, and the considerable overlap with other databases (i.e. the number of duplicates), we limited our search to the three most commonly searched databases in previous reviews [20–24, 41, 60]. By limiting our search, it is possible that a few otherwise eligible studies were missed. It is also possible that including other databases (e.g. EMBASE, Dissertation Abstracts International) would have slightly increased the proportion of non-U.S. based interventions.

## Conclusions

Despite limitations, this study used a novel approach to synthesize and profile the recent literature on family-based childhood obesity prevention interventions. Results demonstrate the current emphasis in interventions, and lack of adequate representation of various groups. More interventions that recruit diverse populations, and target behaviors beyond diet and physical activity, are needed to better understand the influence of these characteristics when designing and implementing family-based childhood obesity prevention interventions. The results of this study can be used to inform decision-making around intervention design and funding aimed at filling gaps in the knowledge base. Filling these gaps will lead to a better understanding of how best to target a wide range of behaviors in diverse populations.

## Additional files

Additional file 1: Full search strategy for PubMed database to identify eligible family-based childhood obesity prevention interventions published between 2008 and 2015. (DOCX 135 kb)

Additional file 2: List of eligible articles published between 2008 and 2015 detailing a family-based childhood obesity prevention intervention. (DOCX 210 kb)

Additional file 3: Table S1. Intervention characteristics of family-based childhood obesity prevention interventions separating studies with evaluations from protocols. (DOCX 116 kb)

## Abbreviations

IOM: Institute of Medicine
PRISMA: Preferred Reporting Items for Systematic Reviews and Meta-Analysis
SES: Socioeconomic status
U.S: United States
