# Supplementary material for: Family-based childhood obesity prevention interventions: a systematic review and quantitative content analysis
Source: Int J Behav Nutr Phys Act. 2017 Aug 24;14:113. doi: 10.1186/s12966-017-0571-2 (PMC5571569; doi:10.1186/s12966-017-0571-2)
Supplement: Supplementary file 1 — Full search strategy for PubMed database to identify eligible family-based childhood obesity prevention interventions published between 2008 and 2015. (DOCX 135 kb) [file 12966_2017_571_MOESM1_ESM.docx]

| ("Overweight"[Mesh] OR "Pediatric Obesity"[Mesh] OR "Body Mass Index"[Mesh] OR "Body Weight"[Mesh:NoExp] OR "Ideal Body Weight"[Mesh] OR "Weight Loss"[Mesh:NoExp] OR "Weight Gain"[Mesh] OR "Weight Reduction Programs"[Mesh] OR obesity[tw] OR obese[tw] OR over weight[tw] OR overweight[tw] OR body weight[tw] OR body mass[tw] OR weight status[tw] OR weight for length[tw] OR weight maintenance[tw] OR healthy weight[tw] OR weight los*[tw] OR weight gain*[tw] OR weight reduction[tw] OR weight percentile[tw] OR adiposity[tw] OR body fat[tw]  OR fatness[tw])  AND  (Infant[Mesh] OR Child[Mesh] OR "Adolescent"[Mesh]  OR infant[tw] OR infants[tw] OR infant’s[tw] OR infancy[tw] OR neonate[tw] OR neonates[tw] OR neonatal[tw] OR newborn[tw] OR newborns[tw] OR newborn’s[tw] OR new born*[tw] OR baby[tw] OR babies[tw] OR preschooler*[tw] OR pre schooler*[tw] OR pre school[tw] OR preschool[tw] OR toddler*[tw] OR child[tw] OR children[tw] OR children’s[tw] OR childhood[tw] OR boy[tw] OR boys[tw] OR girl[tw] OR girls[tw] OR youth[tw] OR youths[tw] OR youth's[tw] OR adolescent[tw] OR adolescents[tw] OR adolescence[tw] OR teen[tw] OR teens[tw] OR teenager[tw] OR teenagers[tw] OR teenage[tw] OR schoolchild*[tw] OR school age*[tw] OR schoolage*[tw] OR nursery school*[tw]  OR kindergar*[tw] OR primary school*[tw] OR secondary school*[tw] OR grade school*[tw] OR elementary school*[tw]  OR elementary student*[tw] OR high school*[tw] OR highschool*[tw] OR pediatric[tw] OR paediatric[tw])  AND  ("Overweight"[Mesh] OR "Pediatric Obesity"[Mesh] OR "Body Mass Index"[Mesh] OR "Body Weight"[Mesh:NoExp] OR "Ideal Body Weight"[Mesh] OR "Weight Loss"[Mesh:NoExp] OR "Weight Gain"[Mesh] OR "Weight Reduction Programs"[Mesh] OR obesity[tw] OR obese[tw] OR over weight[tw] OR overweight[tw] OR body weight[tw] OR body mass[tw] OR weight status[tw] OR weight for length[tw] OR weight maintenance[tw] OR healthy weight[tw] OR weight los*[tw] OR weight gain*[tw] OR weight reduction[tw] OR weight percentile[tw] OR adiposity[tw] OR body fat[tw]  OR fatness[tw]) AND ("Parents"[Mesh] OR parent[tw] OR parents[tw] OR mother[tw] OR mothers[tw] OR father[tw] OR fathers[tw] OR primary care giver*[tw] OR primary caregiver*[tw] OR home based[tw] OR in the home[tw] OR at home[tw] OR family[tw] OR families[tw] AND (Infant[Mesh] OR Child[Mesh] OR "Adolescent"[Mesh]  OR infant[tw] OR infants[tw] OR infant’s[tw] OR infancy[tw] OR neonate[tw] OR neonates[tw] OR neonatal[tw] OR newborn[tw] OR newborns[tw] OR newborn’s[tw] OR new born*[tw] OR baby[tw] OR babies[tw] OR preschooler*[tw] OR pre schooler*[tw] OR pre school[tw] OR preschool[tw] OR toddler*[tw] OR child[tw] OR children[tw] OR children’s[tw] OR childhood[tw] OR boy[tw] OR boys[tw] OR girl[tw] OR girls[tw] OR youth[tw] OR youths[tw] OR youth's[tw] OR adolescent[tw] OR adolescents[tw] OR adolescence[tw] OR teen[tw] OR teens[tw] OR teenager[tw] OR teenagers[tw] OR teenage[tw] OR schoolchild*[tw] OR school age*[tw] OR schoolage*[tw] OR nursery school*[tw]  OR kindergar*[tw] OR primary school*[tw] OR secondary school*[tw] OR grade school*[tw] OR elementary school*[tw]  OR elementary student*[tw] OR high school*[tw] OR highschool*[tw] OR pediatric[tw] OR paediatric[tw]) NOT (comment[ptyp] OR editorial[ptyp] OR letter[ptyp] OR case reports[ptyp] OR news[ptyp] OR review[ptyp]) NOT ("Animals"[Mesh] NOT "Humans"[Mesh]) AND English[lang] AND ("2008/01/01"[PDAT] : "2015/12/31"[PDAT])  AND  "Clinical Trial" [Publication Type] OR "prevention  and  control" [Subheading] OR “therapy” [Subheading:NoExp] OR "Health Education"[Mesh:NoExp] OR "Patient Education as Topic"[Mesh:NoExp] OR "Health Promotion"[Mesh] OR "Counseling"[Mesh] OR "Nutritionists"[Mesh] OR "Nurses"[Mesh:NoExp] OR "Nurses, Community Health"[Mesh] OR "Family Nurse Practitioners"[Mesh] OR "Pediatric Nurse Practitioners"[Mesh] OR trial[tw] OR random*[tw] OR intervention[tw] OR preventive[tw] OR prevention[tw] OR treatment[tw] OR therapy[tw] OR therapeutic[tw] OR counseling[tw] OR counselor*[tw] OR dietitian*[tw] OR nutritionist*[tw] OR nurse[tw] OR nurses[tw] OR promotion[tw] OR promote[tw] OR promoting[tw] OR education*[tw] OR behavior change*[tw] OR behaviour change*[tw] OR behavioral change*[tw] OR behavioural change*[tw] |
| --- |
