# Supplementary material for: Family-based childhood obesity prevention interventions: a systematic review and quantitative content analysis
Source: Int J Behav Nutr Phys Act. 2017 Aug 24;14:113. doi: 10.1186/s12966-017-0571-2 (PMC5571569; doi:10.1186/s12966-017-0571-2)
Supplement: Supplementary file 2 — List of eligible articles published between 2008 and 2015 detailing a family-based childhood obesity prevention intervention. (DOCX 210 kb) [file 12966_2017_571_MOESM2_ESM.docx]

1. Adab, P., Pallan, M. J., Cade, J., Ekelund, U., Barrett, T., Daley, A., … Cheng, K. K. (2014). Preventing childhood obesity, phase II feasibility study focusing on south Asians: BEACHeS. *BMJ Open*, *4*(4), . doi:10.1136/bmjopen-2013-004579
2. Adamo, K. B., Ferraro, Z. M., Goldfield, G., Keely, E., Stacey, D., Hadjiyannakis, S., … Barrowman, N. J. (2013). The maternal obesity management (MOM) trial protocol: A lifestyle intervention during pregnancy to minimize downstream obesity. *Contemporary Clinical Trials*, *35*(1), 87–96. doi:10.1016/j.cct.2013.02.010
3. Adams, A. K., LaRowe, T. L., Cronin, K. A., Prince, R. J., Wubben, D. P., Parker, T., & Jobe, J. B. (2012). The healthy children, strong families intervention: Design and community participation. *The Journal of Primary Prevention*, *33*(4), 175–185. doi:10.1007/s10935-012-0275-y
4. Avis, J. L., Cave, A. L., Donaldson, S., Ellendt, C., Holt, N. L., Jelinski, S., … Ball, G. D. (2015). Working with parents to prevent childhood obesity: Protocol for a primary care-based eHealth study. *JMIR Research Protocols*, *4*(1), e35. doi:10.2196/resprot.4147
5. Ayala, G. X., Elder, J. P., Campbell, N. R., Arredondo, E., Baquero, B., Crespo, N. C., & Slymen, D. J. (2010). Longitudinal intervention effects on parenting of the Aventuras para Niños study. *American Journal of Preventive Medicine*, *38*(2), 154–162. doi:10.1016/j.amepre.2009.09.038
6. Bacardí-Gascon, M., Pérez-Morales, M., & Jiménez-Cruz, A. (2012). A six month randomized school intervention and an 18-month follow-up intervention to prevent childhood obesity in Mexican elementary schools. *Nutrición Hospitalaria*, *3*(27), 755-762.
7. Barber, S. E., Jackson, C., Akhtar, S., Bingham, D. D., Ainsworth, H., Hewitt, C., … Wright, J. (2013). “Pre-schoolers in the playground” an outdoor physical activity intervention for children aged 18 months to 4 years old: Study protocol for a pilot cluster randomised controlled trial. *Trials*, *14*(1), 326. doi:10.1186/1745-6215-14-326
8. Barkin, S. L., Gesell, S. B., Po’e, E. K., Escarfuller, J., & Tempesti, T. (2012). Culturally tailored, family-centered, behavioral obesity intervention for Latino-American preschool-aged children. *PEDIATRICS*, *130*(3), 445–456. doi:10.1542/peds.2011-3762
9. Barnes, A. T., Plotnikoff, R. C., Collins, C. E., & Morgan, P. J. (2015) Feasibility and Preliminary Efficacy of the MADE4Life Program: A Pilot Randomized Controlled Trial. *Journal of Physical Activity and Health*, 12, 1378-1393.
10. Bender, M. S., Clark, M. J., & Gahagan, S. (2014). Community engagement approach: Developing a culturally appropriate intervention for Hispanic mother-child Dyads. *Journal of Transcultural Nursing*, *25*(4), 373–382. doi:10.1177/1043659614523473
11. Bender, M. S., Nader, P. R., Kennedy, C., & Gahagan, S. (2013). A culturally appropriate intervention to improve health behaviors in Hispanic Mother–Child Dyads. *Childhood Obesity*, *9*(2), 157–163. doi:10.1089/chi.2012.0118
12. Berry, D. C., McMurray, R., Schwartz, T. A., Skelly, A., Sanchez, M., Neal, M., & Hall, G. (2012). Rationale, design, methodology and sample characteristics for the family partners for health study: A cluster randomized controlled study. *BMC Public Health*, *12*(1), . doi:10.1186/1471-2458-12-250
13. Birken, C. S., Maguire, J., Mekky, M., Manlhiot, C., Beck, C. E., DeGroot, J., … Parkin, P. C. (2012). Office-based Randomized controlled trial to reduce screen time in preschool children. *PEDIATRICS*, *130*(6), 1110–1115. doi:10.1542/peds.2011-3088
14. Bonuck, K., Avraham, S. B., Lo, Y., Kahn, R., & Hyden, C. (2014). Bottle-weaning intervention and toddler overweight. *The Journal of Pediatrics*, *164*(2), 306–312.e2. doi:10.1016/j.jpeds.2013.09.029
15. Bruss, M. B., Michael, T. J., Morris, J. R., Applegate, B., Dannison, L., Quitugua, J. A., … Klein, D. J. (2010). Childhood obesity prevention: An intervention targeting primary caregivers of school children. *Obesity*, *18*(1), 99–107. doi:10.1038/oby.2009.111
16. Burnet, D. L., Plaut, A. J., Wolf, S. A., Huo, D., Solomon, M. C., Dekayie, G., … Chin, M. H. (2011). Reach-out: A family-based diabetes prevention program for African American youth. *Journal of the National Medical Association*, *103*(3), 269–277. doi:10.1016/s0027-9684(15)30290-x
17. Burrows, T., Bray, J., Morgan, P. J., & Collins, C. (2013). Pilot intervention in an economically disadvantaged community: The back‐to‐basics after‐school healthy lifestyle program. *Nutrition & Dietetics*, *70*(4), 270-277.
18. Burrows, T. L., Lucas H., Morgan, P. J., Bray, J., & Collins, C. E. (2015) Impact Evaluation of an After-school Cooking Skills Program in a Disadvantaged Community: Back to Basics. *Canadian Journal of Dietetic Practice and Research,* 76(3), 126-132.
19. Cameron, A. J., Ball, K., Hesketh, K. D., McNaughton, S. A., Salmon, J., Crawford, D. A., … Campbell, K. J. (2014). Variation in outcomes of the Melbourne infant, feeding, activity and nutrition trial (inFANT) program according to maternal education and age. *Preventive Medicine*, *58*, 58–63. doi:10.1016/j.ypmed.2013.10.021
20. Campbell, K. J., Lioret, S., McNaughton, S. A., Crawford, D. A., Salmon, J., Ball, K., … Hesketh, K. D. (2013). A parent-focused intervention to reduce infant obesity risk behaviors: A Randomized trial. *PEDIATRICS*, *131*(4), 652–660. doi:10.1542/peds.2012-2576
21. Campbell, K., Hesketh, K., Crawford, D., Salmon, J., Ball, K., & McCallum, Z. (2008). The infant feeding activity and nutrition trial (INFANT) an early intervention to prevent childhood obesity: Cluster-randomised controlled trial. *BMC Public Health*, *8*(1), 103. doi:10.1186/1471-2458-8-103
22. Carlsen, E. M., Kyhnaeb, A., Renault, K. M., Cortes, D., Michaelsen, K. F., & Pryds, O. (2013). Telephone-based support prolongs breastfeeding duration in obese women: A randomized trial. *American Journal of Clinical Nutrition*, *98*(5), 1226–1232. doi:10.3945/ajcn.113.059600
23. Castro, D. C., Samuels, M., & Harman, A. E. (2013). Growing healthy kids. *American Journal of Preventive Medicine*, *44*(3), S193–S199. doi:10.1016/j.amepre.2012.11.024
24. Catenacci, V., Barrett, C., Odgen, L., Browning, R., Schaefer, C. A., Hill, J., & Wyatt, H. (2014). Changes in physical activity and sedentary behavior in a Randomized trial of an Internet-Based versus Workbook-Based family intervention study. *Journal of Physical Activity and Health*, *11*(2), 348–358. doi:10.1123/jpah.2012-0043
25. Centis, E., Marzocchi, R., Di Luzio, R., Moscatiello, S., Salardi, S., Villanova, N., & Marchesini, G. (2012). A controlled, class-based multicomponent intervention to promote healthy lifestyle and to reduce the burden of childhood obesity. *Pediatric Obesity*, *7*(6), 436–445. doi:10.1111/j.2047-6310.2012.00079.x
26. Chen, J. L., Weiss, S., Heyman, M. B., & Lustig, R. H. (2009). Efficacy of a child-centred and family-based program in promoting healthy weight and healthy behaviors in Chinese American children: A randomized controlled study. *Journal of Public Health*, *32*(2), 219–229. doi:10.1093/pubmed/fdp105
27. Chen, J.-L., Weiss, S. J., Heyman, M. B., Cooper, B., & Lustig, R. H. (2010). ABC program for improving coping and quality of life in Chinese-American children. *Nursing Research*, *59*(4), 270–279. http://doi.org/10.1097/NNR.0b013e3181e507c7
28. Chen, J.-L., Weiss, S., Heyman, M. B., Cooper, B., & Lustig, R. H. (2011). The efficacy of the web-based childhood obesity prevention program in Chinese American adolescents (web ABC study). *Journal of Adolescent Health*, *49*(2), 148–154. doi:10.1016/j.jadohealth.2010.11.243
29. Cloutier, M. M., Wiley, J., Huedo-Medina, T., Ohannessian, C. M., Grant, A., Hernandez, D., & Gorin, A. A. (2015). Outcomes from a pediatric primary care weight management program: Steps to growing up healthy. *The Journal of Pediatrics*, *167*(2), 372–377.e1. doi:10.1016/j.jpeds.2015.05.028
30. Cloutier, M. M., Wiley, J., Wang, Z., Grant, A., & Gorin, A. A. (2015). The early childhood obesity prevention program (ECHO): An ecologically-based intervention delivered by home visitors for newborns and their mothers. *BMC Public Health*, *15*(1), 584. doi:10.1186/s12889-015-1897-9
31. Coleman, K. J., Ocana, L. L., Walker, C., Araujo, R. A., Gutierrez, V., Shordon, M., … Philis-Tsimikas, A. (2010). Outcomes from a culturally tailored diabetes prevention program in Hispanic families from a low-income school: Horton hawks stay healthy (HHSH). *The Diabetes Educator*, *36*(5), 784–792. doi:10.1177/0145721710377360
32. Crespo, N. C., Elder, J. P., Ayala, G. X., Slymen, D. J., Campbell, N. R., Sallis, J. F., … Arredondo, E. M. (2012). Results of a Multi-level Intervention to Prevent and Control Childhood Obesity among Latino Children: The Aventuras Para Niños Study. *Annals of Behavioral Medicine : A Publication of the Society of Behavioral Medicine*, *43*(1), 84–100. http://doi.org/10.1007/s12160-011-9332-7
33. Daniels, L. A., Magarey, A., Battistutta, D., Nicholson, J. M., Farrell, A., Davidson, G., & Cleghorn, G. (2009). The NOURISH randomised control trial: Positive feeding practices and food preferences in early childhood - a primary prevention program for childhood obesity. *BMC Public Health*, *9*(1), . doi:10.1186/1471-2458-9-387
34. Daniels, L. A., Mallan, K. M., Battistutta, D., Nicholson, J. M., Perry, R., & Magarey, A. (2012). Evaluation of an intervention to promote protective infant feeding practices to prevent childhood obesity: Outcomes of the NOURISH RCT at 14 months of age and 6 months post the first of two intervention modules. *International Journal of Obesity*, *36*(10), 1292–1298. doi:10.1038/ijo.2012.96
35. Daniels, L. A., Mallan, K. M., Battistutta, D., Nicholson, J. M., Meedeniya, J. E., Bayer, J. K., & Magarey, A. (2014). Child eating behavior outcomes of an early feeding intervention to reduce risk indicators for child obesity: The NOURISH RCT. *Obesity*, *22*(5), E104–E111. doi:10.1002/oby.20693
36. Daniels, L. A., Mallan, K. M., Nicholson, J. M., Battistutta, D., & Magarey, A. (2013). Outcomes of an early feeding practices intervention to prevent childhood obesity. *PEDIATRICS*, *132*(1), e109–e118. doi:10.1542/peds.2012-2882
37. Daniels, L. A., Mallan, K. M., Nicholson, J. M., Thorpe, K., Nambiar, S., Mauch, C. E., & Magarey, A. (2015). An early feeding practices intervention for obesity prevention. *PEDIATRICS*, *136*(1), e40–e49. doi:10.1542/peds.2014-4108
38. Davis, A. M., Gallagher, K., Taylor, M., Canter, K., Gillette, M. D., Wambach, K., & Nelson, E.-L. (2013). An in-home intervention to improve nutrition, physical activity, and knowledge among low-income teen mothers and their children. *Journal of Developmental & Behavioral Pediatrics*, *34*(8), 609–615. doi:10.1097/dbp.0b013e3182a509df
39. Davison, K. K., Jurkowski, J. M., Li, K., Kranz, S., & Lawson, H. A. (2013). A childhood obesity intervention developed by families for families: Results from a pilot study. *International Journal of Behavioral Nutrition and Physical Activity*, *10*(1), 3. doi:10.1186/1479-5868-10-3
40. Dawson-McClure, S., Brotman, L. M., Theise, R., Palamar, J. J., Kamboukos, D., Barajas, R. G., & Calzada, E. J. (2014). Early childhood obesity prevention in low-income, urban communities. *Journal of Prevention & Intervention in the Community*, *42*(2), 152–166. doi:10.1080/10852352.2014.881194
41. De Bock, F., Breitenstein, L., & Fischer, J. E. (2011). Positive impact of a pre-school-based nutritional intervention on children’s fruit and vegetable intake: Results of a cluster-randomized trial. *Public Health Nutrition*, *15*(03), 466–475. doi:10.1017/s136898001100200x
42. De Bock, F., Fischer, J. E., Hoffmann, K., & Renz-Polster, H. (2010). A participatory parent-focused intervention promoting physical activity in preschools: Design of a cluster-randomized trial. *BMC Public Health*, *10*(1), . doi:10.1186/1471-2458-10-49
43. De Bock, F., Genser, B., Raat, H., Fischer, J. E., & Renz-Polster, H. (2013). A participatory physical activity intervention in Preschools. *American Journal of Preventive Medicine*, *45*(1), 64–74. doi:10.1016/j.amepre.2013.01.032
44. de la Torre, A., Sadeghi, B., Green, R. D., Kaiser, L. L., Flores, Y. G., Jackson, C. F., … Schaefer, S. E. (2013). Niños Sanos, Familia sana: Mexican immigrant study protocol for a multifaceted CBPR intervention to combat childhood obesity in two rural California towns. *BMC Public Health*, *13*(1). doi:10.1186/1471-2458-13-1033
45. de Vries, A., Huiting, H., van den Heuvel, E., L’Abée, C., Corpeleijn, E., & Stolk, R. (2015). An activity stimulation programme during a child’s first year reduces some indicators of adiposity at the age of two-and-a-half. *Acta Paediatrica*, *104*(4), 414–421. doi:10.1111/apa.12880
46. Delisle, C., Sandin, S., Forsum, E., Henriksson, H., Trolle-Lagerros, Y., Larsson, C., … Löf, M. (2015). A web- and mobile phone-based intervention to prevent obesity in 4-year-olds (MINISTOP): A population-based randomized controlled trial. *BMC Public Health*, *15*(1). doi:10.1186/s12889-015-1444-8
47. Denney-Wilson, E., Laws, R., Russell, C.G., Ong, K., Taki, S., Elliot R., … Campbell, K. J. (2015) Preventing obesity in infants: the Growing healthy feasibility trial protocol. *BMJ Open*, 5, 1-12. doi:10.1136/bmjopen-2015-009258
48. Döring, N., Hansson, L. M., Andersson, E., Bohman, B., Westin, M., Magnusson, M., … Rasmussen, F. (2014). Primary prevention of childhood obesity through counselling sessions at Swedish child health centres: Design, methods and baseline sample characteristics of the PRIMROSE cluster-randomised trial. *BMC Public Health*, *14*(1), 335. doi:10.1186/1471-2458-14-335
49. Dulin Keita, A., Risica, P. M., Drenner, K. L., Adams, I., Gorham, G., & Gans, K. M. (2014). Feasibility and acceptability of an early childhood obesity prevention intervention: Results from the healthy homes, healthy families pilot study. *Journal of Obesity*, *2014*, 1–16. doi:10.1155/2014/378501
50. Duncanson, K., Burrows, T., & Collins, C. (2012). Study protocol of a parent-focused child feeding and dietary intake intervention: The feeding healthy food to kids randomised controlled trial. *BMC Public Health*, *12*(1), 564. doi:10.1186/1471-2458-12-564
51. Elder, J. P., Crespo, N. C., Corder, K., Ayala, G. X., Slymen, D. J., Lopez, N. V., … McKenzie, T. L. (2013). Childhood obesity prevention and control in city recreation centres and family homes: The MOVE/me Muevo project. *Pediatric Obesity*, *9*(3), 218–231. doi:10.1111/j.2047-6310.2013.00164.x
52. Eneli, I. U., Tylka, T. L., Hummel, J., Watowicz, R. P., Perez, S. A., Kaciroti, N., & Lumeng, J. C. (2015). Rationale and design of the feeding dynamic intervention (FDI) study for self-regulation of energy intake in preschoolers. *Contemporary Clinical Trials*, *41*, 325–334. doi:10.1016/j.cct.2015.01.006
53. Escobar-Chaves, S.L., Markham, C. M., Addy, R. C., Greisinger, A., Murray, N. G., & Brehm, B. (2010). The fun families study: Intervention to reduce children’s TV viewing. *Obesity*, *18*(n1s), S99–S101. doi:10.1038/oby.2009.438
54. Falbe, J., Cadiz, A. A., Tantoco, N. K., Thompson, H. R., & Madsen, K. A. (2015). Active and healthy families: A Randomized controlled trial of a culturally tailored obesity intervention for Latino children. *Academic Pediatrics*, *15*(4), 386–395. doi:10.1016/j.acap.2015.02.004
55. Fangupo, L. J., Heath, A. M., Williams, S. M., Somerville, M. R., Lawrence, J. A., Gray, A. R., … Taylor, R. W. (2015) Impact of an early-life intervention on the nutrition behaviors of 2-y-old children: a randomized controlled trial. *America Journal of Clinical Nutrition,* 102, 704-712.
56. Fitzgibbon, M. L., Stolley, M. R., Schiffer, L., Kong, A., Braunschweig, C. L., Gomez-Perez, S. L., … Dyer, A. R. (2013). Family-based hip-hop to health: Outcome results. *Obesity*, *21*(2), 274–283. doi:10.1002/oby.20269
57. Flattum, C., Draxten, M., Horning, M., Fulkerson, J. A., Neumark-Sztainer, D., Garwick, A., … Story, M. (2015). HOME plus: Program design and implementation of a family-focused, community-based intervention to promote the frequency and healthfulness of family meals, reduce children’s sedentary behavior, and prevent obesity. *International Journal of Behavioral Nutrition and Physical Activity*, *12*(1), . doi:10.1186/s12966-015-0211-7
58. French, G. M., Nicholson, L., Skybo, T., Klein, E. G., Schwirian, P. M., Murray-Johnson, L., … Groner, J. A. (2012). An evaluation of mother-centered anticipatory guidance to reduce Obesogenic infant feeding behaviors. *PEDIATRICS*, *130*(3), e507–e517. doi:10.1542/peds.2011-3027
59. French, S. A., Gerlach, A. F., Mitchell, N. R., Hannan, P. J., & Welsh, E. M. (2011). Household obesity prevention: Take Action—a Group-Randomized trial. *Obesity*, *19*(10), 2082–2088. doi:10.1038/oby.2010.328
60. Frenn, M., Pruszynski, J. E., Felzer, H., & Zhang, J. (2013). Authoritative feeding behaviors to reduce child BMI through online interventions. *Journal for Specialists in Pediatric Nursing*, *18*(1), 65–77. doi:10.1111/jspn.12008
61. Fulkerson, J. A., Friend, S., Flattum, C., Horning, M., Draxton, M., Neumark-Sztainer, D., … Kubik, M.Y. (2015) *International Journal of Behavioral Nutrition and Physical Activity*, 12(154). doi:10.1186/s12966-015-0320-3
62. Fulkerson, J. A., Neumark-Sztainer, D., Story, M., Gurvich, O., Kubik, M. Y., Garwick, A., & Dudovitz, B. (2014). The Healthy Home Offerings via the Mealtime Environment (HOME) Plus study: Design and methods. *Contemporary Clinical Trials*, *38*(1), 59–68. http://doi.org/10.1016/j.cct.2014.01.006
63. Fulkerson, J. A., Rydell, S., Kubik, M. Y., Lytle, L., Boutelle, K., Story, M., … Garwick, A. (2010). Healthy home offerings via the mealtime environment (HOME): Feasibility, acceptability, and outcomes of a pilot study. *Obesity*, *18*(n1s), S69–S74. doi:10.1038/oby.2009.434
64. Gorin, A. A., Wiley, J., Ohannessian, C. M., Hernandez, D., Grant, A., & Cloutier, M. M. (2014). Steps to growing up healthy: A pediatric primary care based obesity prevention program for young children. *BMC Public Health*, *14*(1), . doi:10.1186/1471-2458-14-72
65. GreenMills, L. L., Davison, K. K., Gordon, K. E., Li, K., & Jurkowski, J. M. (2013). Evaluation of a childhood obesity awareness campaign targeting head start families: Designed by parents for parents. *Journal of Health Care for the Poor and Underserved*, *24*(2A), 25–33. doi:10.1353/hpu.2013.0096
66. Groner, J. A., Skybo, T., Murray-Johnson, L., Schwirian, P., Eneli, I., Sternstein, A., … French, G. (2009). Anticipatory guidance for prevention of childhood obesity: Design of the MOMS project. *Clinical Pediatrics*, *48*(5), 483–492. doi:10.1177/0009922809331799
67. Haines, J., McDonald, J., O’Brien, A., Sherry, B., Bottino, C. J., Schmidt, M. E., & Taveras, E. M. (2013). Healthy habits, happy homes: randomized trial to improve household routines for obesity prevention among preschool-aged children. *JAMA pediatrics*, *167*(11), 1072-1079.
68. Haire-Joshu, D., Elliott, M. B., Caito, N. M., Hessler, K., Nanney, M. S., Hale, N., … Brownson, R. C. (2008). High 5 for kids: The impact of a home visiting program on fruit and vegetable intake of parents and their preschool children. *Preventive Medicine*, *47*(1), 77–82. doi:10.1016/j.ypmed.2008.03.016
69. Hammons, A. J., Wiley, A. R., Fiese, B. H., & Teran-Garcia, M. (2013). Six-Week Latino family prevention pilot program effectively promotes healthy behaviors and reduces Obesogenic behaviors. *Journal of Nutrition Education and Behavior*, *45*(6), 745–750. doi:10.1016/j.jneb.2013.01.023
70. Hannon, T. S., Carroll, A. E., Palmer, K. N., Saha, C., Childers, W. K., & Marrero, D. G. (2015). Rationale and design of a comparative effectiveness trial to prevent type 2 diabetes in mothers and children: The ENCOURAGE healthy families study. *Contemporary Clinical Trials*, *40*, 105–111. doi:10.1016/j.cct.2014.11.016
71. Hardman, C. A., Horne, P. J., & Lowe, C. F. (2009). A home-based intervention to increase physical activity in girls: the Fit ‘n’Fun Dudes program. *Journal of Exercise Science & Fitness*, *7*(1), 1-8.
72. Hesketh, K. D., Campbell, K., Salmon, J., McNaughton, S. A., McCallum, Z., Cameron, A., … Crawford, D. (2013). The Melbourne infant feeding, activity and nutrition trial (inFANT) program follow-up. *Contemporary Clinical Trials*, *34*(1), 145–151. doi:10.1016/j.cct.2012.10.008
73. Hoelscher, D. M., Butte, N. F., Barlow, S., Vandewater, E. A., Sharma, S. V., Huang, T., … Kelder, S. H. (2015). Incorporating primary and secondary prevention approaches to address childhood obesity prevention and treatment in a low-income, ethnically diverse population: Study design and demographic data from the Texas childhood obesity research demonstration (TX CORD) study. *Childhood Obesity*, *11*(1), 71–91. doi:10.1089/chi.2014.0084
74. Horodynski, M. A., Olson, B., Baker, S., Brophy-Herb, H., Auld, G., Van Egeren, L., … Singleterry, L. (2011). Healthy babies through infant-centered feeding protocol: An intervention targeting early childhood obesity in vulnerable populations. *BMC Public Health*, *11*(1), . doi:10.1186/1471-2458-11-868
75. Horodynski, M. A., Silk, K., Hsieh, G., Hoffman, A., & Robson, M. (2015). Tools for teen moms to reduce infant obesity: A randomized clinical trial. *BMC Public Health*, *15*(1), 22. doi:10.1186/s12889-015-1345-x
76. Horton, L. A., Parada, H., Slymen, D. J., Arredondo, E., Ibarra, L., & Ayala, G. X. (2013). Targeting children's dietary behaviors in a family intervention:'Entre familia: reflejos de salud'. *salud pública de méxico*, *55*, 397-405.
77. Jacobson, D., & Melnyk, B. M. (2012). A primary care healthy choices intervention program for overweight and obese school-age children and their parents. *Journal of Pediatric Health Care*, *26*(2), 126–138. doi:10.1016/j.pedhc.2010.07.004
78. Jones, R., Wells, M., Okely, A., Lockyer, L., & Walton, K. (2011). Is an online healthy lifestyles program acceptable for parents of preschool children?. *Nutrition & Dietetics*, *68*(2), 149-154.
79. Junnila, R., Aromaa, M., Heinonen, O. J., Lagström, H., Liuksila, P.-R., Vahlberg, T., & Salanterä, S. (2012). The weighty matter intervention: A family-centered way to tackle an overweight childhood. *Journal of Community Health Nursing*, *29*(1), 39–52. doi:10.1080/07370016.2012.645742
80. Kaiser, L., Martinez, J., Horowitz, M., Lamp, C., Johns, M., Espinoza, D., ... de la Torre, A. (2015). Adaptation of a Culturally Relevant Nutrition and Physical Activity Program for Low-Income, Mexican-Origin Parents With Young Children. *Preventing Chronic Disease*, *12*, E72.
81. Karanja, N., Aickin, M., Lutz, T., Mist, S., Jobe, J. B., Maupomé, G., & Ritenbaugh, C. (2012). A Community-Based Intervention to Prevent Obesity Beginning at Birth among American Indian Children: Study Design and Rationale for the PTOTS study. *The Journal of Primary Prevention*, *33*(4), 161–174. http://doi.org/10.1007/s10935-012-0278-8
82. Karanja, N., Lutz, T., Ritenbaugh, C., Maupome, G., Jones, J., Becker, T., & Aickin, M. (2010). The TOTS Community Intervention to Prevent Overweight in American Indian Toddlers: A Feasibility and Efficacy Study. *Journal of Community Health*, *35*(6), 667–675. http://doi.org/10.1007/s10900-010-9270-5
83. Kargarfard, M., Kelishadi, R., Ziaee, V., Ardalan, G., Halabchi, F., Mazaheri, R., … Hayatbakhsh, M. R. (2012). The impact of an after-school physical activity program on health-related fitness of mother/daughter pairs: CASPIAN study. *Preventive Medicine*, *54*(3-4), 219–223. doi:10.1016/j.ypmed.2012.01.010
84. Kavanagh, K. F., Cohen, R. J., Heinig, M. J., & Dewey, K. G. (2008). Educational intervention to modify bottle-feeding behaviors among formula-feeding mothers in the WIC program: Impact on infant formula intake and weight gain. *Journal of Nutrition Education and Behavior*, *40*(4), 244–250. doi:10.1016/j.jneb.2007.01.002
85. Kelishadi, R., Ziaee, V., Ardalan, G., Namazi, A., Noormohammadpour, P., Ghayour-Mobarhan, M., … Poursafa, P. (2010). A National Experience on Physical Activity Initiatives for Adolescent Girls and their Mothers: CASPIAN Study. *Iranian Journal of Pediatrics*, *20*(4), 420–426.
86. Klesges, R. C., Obarzanek, E., Klesges, L. M., Stockton, M. B., Beech, B. M., Murray, D. M., … Sherrill-Mittleman, D. A. (2008). Memphis girls health enrichment multi-site studies (GEMS). *Contemporary Clinical Trials*, *29*(1), 42–55. doi:10.1016/j.cct.2007.05.001
87. Knowlden, A. P., Sharma, M., Cottrell, R. R., Wilson, B. R. A., & Johnson, M. L. (2014). Impact evaluation of enabling mothers to prevent pediatric obesity through web-based education and reciprocal determinism (EMPOWER) Randomized control trial. *Health Education & Behavior*, *42*(2), 171–184. doi:10.1177/1090198114547816
88. Knowlden, A., & Sharma, M. (2012). A feasibility and efficacy Randomized controlled trial of an online preventative program for childhood obesity: Protocol for the EMPOWER intervention. *JMIR Research Protocols*, *1*(1), e5. doi:10.2196/resprot.2141
89. Koulouglioti, C., Cole, R., McQuillan, B., Moskow, M., Kueppers, J., & Pigeon, W. (2013). Feasibility of an individualized, home-based obesity prevention program for preschool-age children. *Children’s Health Care*, *42*(2), 134–152. doi:10.1080/02739615.2013.766099
90. Lakshman, R., Whittle, F., Hardeman, W., Suhrcke, M., Wilson, E., Griffin, S., & Ong, K. K. (2015) Effectiveness of a behavioural intervention to prevent excessive weight gain during infancy (The Baby Milk Trial): study protocol for a randomised controlled trial. *Trials*, 16(442). doi:10.1186/s13063-015-0941-5
91. Larsen, K. T., Huang, T., Møller, N. C., Andersen, L. B., & Ried-Larsen, M. (2014). Effectiveness of a one-year multi-component day-camp intervention for overweight children: Study protocol of the Odense overweight intervention study (OOIS). *BMC Public Health*, *14*(1), . doi:10.1186/1471-2458-14-313
92. Lloyd, A. B., Lubans, D. R., Plotnikoff, R. C., & Morgan, P. J. (2015). Paternal lifestyle-related parenting practices mediate changes in children’s dietary and physical activity behaviors: Findings from the healthy Dads, healthy kids community Randomized controlled trial. *Journal of Physical Activity and Health*, *12*(9), 1327–1335. doi:10.1123/jpah.2014-0367
93. Lloyd, A. B., Lubans, D. R., Plotnikoff, R. C., & Morgan, P. J. (2014). Impact of the “healthy Dads, healthy kids” lifestyle programme on the activity- and diet-related parenting practices of fathers and mothers. *Pediatric Obesity*, *9*(6), e149–e155. doi:10.1111/ijpo.248
94. Louzada, M. L. d. C., Campagnolo, P. D. B., Rauber, F., & Vitolo, M. R. (2012). Long-term effectiveness of maternal dietary counseling in a low-income population: A Randomized field trial. *PEDIATRICS*, *129*(6), e1477–e1484. doi:10.1542/peds.2011-3063
95. Lubans, D. R., Morgan, P. J., Collins, C. E., Okely, A. D., Burrows, T., & Callister, R. (2012). Mediators of weight loss in the “Healthy Dads, Healthy Kids” pilot study for overweight fathers. *The International Journal of Behavioral Nutrition and Physical Activity*, *9*, 45. http://doi.org/10.1186/1479-5868-9-45
96. Lynch, W. C., Martz, J., Eldridge, G., Bailey, S. J., Benke, C., & Paul, L. (2012). Childhood obesity prevention in rural settings: Background, rationale, and study design of “4-Health,” a parent-only intervention. *BMC Public Health*, *12*(1), 255. doi:10.1186/1471-2458-12-255
97. Martínez-Andrade, G. O., Cespedes, E. M., Rifas-Shiman, S. L., Romero-Quechol, G., González-Unzaga, M. A., Benítez-Trejo, M. A., … Gillman, M. W. (2014). Feasibility and impact of Creciendo Sanos, a clinic-based pilot intervention to prevent obesity among preschool children in Mexico city. *BMC Pediatrics*, *14*(1), . doi:10.1186/1471-2431-14-77
98. McGowan, L., Cooke, L. J., Gardner, B., Beeken, R. J., Croker, H., & Wardle, J. (2013). Healthy feeding habits: Efficacy results from a cluster-randomized, controlled exploratory trial of a novel, habit-based intervention with parents. *American Journal of Clinical Nutrition*, *98*(3), 769–777. doi:10.3945/ajcn.112.052159
99. Mihas, C., Mariolis, A., Manios, Y., Naska, A., Arapaki, A., Mariolis-Sapsakos, T., & Tountas, Y. (2009). Evaluation of a nutrition intervention in adolescents of an urban area in Greece: Short- and long-term effects of the VYRONAS study. *Public Health Nutrition*, *13*(05), 712. doi:10.1017/s1368980009991625
100. Miller, A. L., Horodynski, M. A., Herb, H. E. B., Peterson, K. E., Contreras, D., Kaciroti, N., … Lumeng, J. C. (2012). Enhancing self-regulation as a strategy for obesity prevention in head start preschoolers: The growing healthy study. *BMC Public Health*, *12*(1), . doi:10.1186/1471-2458-12-1040
101. Minossi, V., & Pellanda, L. (2015). The “Happy Heart” educational program for changes in health habits in children and their families: Protocol for a randomized clinical trial. *BMC Pediatrics*, *15*(1), 19. doi:10.1186/s12887-015-0336-5
102. Morgan, P. J., Collins, C. E., Plotnikoff, R. C., Callister, R., Burrows, T., Fletcher, R., … Lubans, D. R. (2014). The “healthy Dads, healthy kids” community randomized controlled trial: A community-based healthy lifestyle program for fathers and their children. *Preventive Medicine*, *61*, 90–99. doi:10.1016/j.ypmed.2013.12.019
103. Morgan, P. J., Lubans, D. R., Callister, R., Okely, A. D., Burrows, T. L., Fletcher, R., & Collins, C. E. (2010). The “healthy Dads, healthy kids” randomized controlled trial: Efficacy of a healthy lifestyle program for overweight fathers and their children. *International Journal of Obesity*, *35*(3), 436–447. doi:10.1038/ijo.2010.151
104. Morgan, P. J., Lubans, D. R., Plotnikoff, R. C., Callister, R., Burrows, T., Fletcher, R., … Collins, C. E. (2011). The “healthy Dads, healthy kids” community effectiveness trial: Study protocol of a community-based healthy lifestyle program for fathers and their children. *BMC Public Health*, *11*(1), 876. doi:10.1186/1471-2458-11-876
105. Mustila, T., Keskinen, P., & Luoto, R. (2012). Behavioral counseling to prevent childhood obesity – study protocol of a pragmatic trial in maternity and child health care. *BMC Pediatrics*, *12*, 93. http://doi.org/10.1186/1471-2431-12-93
106. Mustila, T., Raitanen, J., Keskinen, P., Saari, A., & Luoto, R. (2013). Pragmatic controlled trial to prevent childhood obesity in maternity and child health care clinics: pregnancy and infant weight outcomes (The VACOPP Study). *BMC Pediatrics*, *13*, 80. http://doi.org/10.1186/1471-2431-13-80
107. Mustila, T., Raitanen, J., Keskinen, P., Saari, A., & Luoto, R. (2012). Lifestyle counseling during pregnancy and offspring weight development until four years of age: Follow-up study of a controlled trial. *Journal of Negative Results in BioMedicine*, *11*(1), . doi:10.1186/1477-5751-11-11
108. Natale, R. A., Messiah, S. E., Asfour, L., Uhlhorn, S. B., Delamater, A., & Arheart, K. L. (2014). Role modeling as an early childhood obesity prevention strategy: effect of parents and teachers on preschool children's healthy lifestyle habits. *Journal of Developmental & Behavioral Pediatrics*, *35*(6), 378-387.
109. Nyberg, G., Sundblom, E., Norman, Å., & Elinder, L. S. (2011). A healthy school start - Parental support to promote healthy dietary habits and physical activity in children: Design and evaluation of a cluster-randomised intervention. *BMC Public Health*, *11*, 185. http://doi.org/10.1186/1471-2458-11-185
110. Nyberg, G., Sundblom, E., Norman, Å., Bohman, B., Hagberg, J., & Elinder, L. S. (2015). Effectiveness of a Universal Parental Support Programme to Promote Healthy Dietary Habits and Physical Activity and to Prevent Overweight and Obesity in 6-Year-Old Children: The Healthy School Start Study, a Cluster-Randomised Controlled Trial. *PLoS ONE*, *10*(2), e0116876. http://doi.org/10.1371/journal.pone.0116876
111. Olsen, N. J., Buch-Andersen, T., Händel, M. N., Østergaard, L. M., Pedersen, J., Seeger, C., … Heitmann, B. L. (2012). The healthy start project: A randomized, controlled intervention to prevent overweight among normal weight, preschool children at high risk of future overweight. *BMC Public Health*, *12*(1), . doi:10.1186/1471-2458-12-590
112. Olvera, N., Bush, J. A., Sharma, S. V., Knox, B. B., Scherer, R. L., & Butte, N. F. (2010). BOUNCE: A community-based Mother–daughter healthy lifestyle intervention for low-income Latino families. *Obesity*, *18*(n1s), S102–S104. doi:10.1038/oby.2009.439
113. Olvera, N., Leung, P., Kellam, S. F., Smith, D. W., & Liu, J. (2013). Summer and follow-up interventions to affect Adiposity with mothers and daughters. *American Journal of Preventive Medicine*, *44*(3), S258–S266. doi:10.1016/j.amepre.2012.11.018
114. Olvera, N., Scherer, R., McLeod, J., Graham, M., Knox, B., Hall, K., ... & Bloom, J. (2010). BOUNCE: an exploratory healthy lifestyle summer intervention for girls. *American Journal of Health Behavior*, *34*(2), 144-155.
115. Önnerfält, J., Erlandsson, L.-K., Orban, K., Broberg, M., Helgason, C., & Thorngren-Jerneck, K. (2012). A family-based intervention targeting parents of preschool children with overweight and obesity: Conceptual framework and study design of LOOPS- Lund overweight and obesity preschool study. *BMC Public Health*, *12*(1), . doi:10.1186/1471-2458-12-879
116. Østbye, T., Krause, K. M., Stroo, M., Lovelady, C. A., Evenson, K. R., Peterson, B. L., … Zucker, N. L. (2012). Parent-focused change to prevent obesity in preschoolers: Results from the KAN-DO study. *Preventive Medicine*, *55*(3), 188–195. doi:10.1016/j.ypmed.2012.06.005
117. Østbye, T., Zucker, N. L., Krause, K. M., Lovelady, C. A., Evenson, K. R., Peterson, B. L., … Brouwer, R. J. N. (2011). Kids and adults now! Defeat obesity (KAN-DO): Rationale, design and baseline characteristics. *Contemporary Clinical Trials*, *32*(3), 461–469. doi:10.1016/j.cct.2011.01.017
118. Paineau, D. L., Beaufils, F., Boulier, A., Cassuto, D.-A., Chwalow, J., Combris, P., … Bornet, F. (2008). Family dietary coaching to improve nutritional Intakes and body weight control. *Archives of Pediatrics & Adolescent Medicine*, *162*(1), 34. doi:10.1001/archpediatrics.2007.2
119. Parkinson, K. N., Jones, A. R., Tovee, M. J., Ells, L. J., Pearce, M. S., Araujo-Soares, V., & Adamson, A. J. (2015) A cluster randomised trial testing an intervention to improve parents’ recognition of their child’s weight status: study protocol. *BMC Public Health,*  15(549). doi:10.1186/s12889-015-188-3
120. Parra-Medina, D., Liang, Y., Yin, Z., Esparza, L., & Lopez, L. (2015) Weight Outcomes of Latino Adults and Children Participating in the Y Living Program, a Family-Focused Lifestyle Intervention, San Antonio, 2012-2013. *Preventing Chronic Disease,* 12(E219), 1-13.
121. Paul, I. M., Savage, J. S., Anzman, S. L., Beiler, J. S., Marini, M. E., Stokes, J. L., & Birch, L. L. (2010). Preventing obesity during infancy: A pilot study. *Obesity*, *19*(2), 353–361. doi:10.1038/oby.2010.182
122. Paul, I. M., Williams, J. S., Anzman-Frasca, S., Beiler, J. S., Makova, K. D., Marini, M. E., … Birch, L. L. (2014). The intervention nurses start infants growing on healthy Trajectories (INSIGHT) study. *BMC Pediatrics*, *14*(1), . doi:10.1186/1471-2431-14-184
123. Po’e, E. K., Heerman, W. J., Mistry, R. S., & Barkin, S. L. (2013). Growing right onto wellness (GROW): A family-centered, community-based obesity prevention randomized controlled trial for preschool child–parent pairs. *Contemporary Clinical Trials*, *36*(2), 436–449. doi:10.1016/j.cct.2013.08.013
124. Raat, H., Struijk, M. K., Remmers, T., Vlasblom, E., van Grieken, A., Broeren, S. M., … L’Hoir, M. P. (2013). Primary prevention of overweight in preschool children, the BeeBOFT study (breastfeeding, breakfast daily, outside playing, few sweet drinks, less TV viewing): Design of a cluster randomized controlled trial. *BMC Public Health*, *13*(1), . doi:10.1186/1471-2458-13-974
125. Robinson, T. N., Kraemer, H. C., Matheson, D. M., Obarzanek, E., Wilson, D. M., Haskell, W. L., … Killen, J. D. (2008). Stanford GEMS Phase 2 Obesity Prevention Trial for Low-Income African-American Girls: Design and Sample Baseline Characteristics. *Contemporary Clinical Trials*, *29*(1), 56–69. http://doi.org/10.1016/j.cct.2007.04.007
126. Robinson, T. N., Matheson, D. M., Kraemer, H. C., Wilson, D. M., Obarzanek, E., Thompson, N. S., … Killen, J. D. (2010). A Randomized controlled trial of culturally-tailored dance and reducing screen time to prevent weight gain in low-Income African-American girls: Stanford GEMS. *Archives of Pediatrics & Adolescent Medicine*, *164*(11), 995–1004. http://doi.org/10.1001/archpediatrics.2010.197
127. Ruiter, E. L. M., Fransen, G. A. J., Molleman, G. R. M., van der Velden, K., & Engels, R. C. M. E. (2015). The effectiveness of a web-based Dutch parenting program to prevent overweight in children 9–13 years of age: Study protocol for a two-armed cluster randomized controlled trial. *BMC Public Health*, *15*(1), 148. doi:10.1186/s12889-015-1394-1
128. Sagedal, L. R., Øverby, N. C., Lohne-Seiler, H., Bere, E., Torstveit, M. K., Henriksen, T., & Vistad, I. (2013). Study protocol: Fit for delivery - can a lifestyle intervention in pregnancy result in measurable health benefits for mothers and newborns? A randomized controlled trial. *BMC Public Health*, *13*(1), 132. doi:10.1186/1471-2458-13-132
129. Schroeder, N., Rushovich, B., Bartlett, E., Sharma, S., Gittelsohn, J., & Caballero, B. (2015). Early obesity prevention: A Randomized trial of a practice-based intervention in 0–24-Month infants. *Journal of Obesity*, *2015*, 1–7. doi:10.1155/2015/795859
130. Schwartz, R. P., Vitolins, M. Z., Case, L. D., Armstrong, S. C., Perrin, E. M., Cialone, J., & Bell, R. A. (2012). The YMCA healthy, fit, and strong program: A community-based, family-centered, low-cost obesity prevention/treatment pilot study. *Childhood Obesity*, *8*(6), 577–582. doi:10.1089/chi.2012.0060
131. Sherwood, N. E., French, S. A., Veblen-Mortenson, S., Crain, A. L., Berge, J., Kunin-Batson, A., … Senso, M. (2013). NET-works: Linking families, communities and primary care to prevent obesity in preschool-age children. *Contemporary Clinical Trials*, *36*(2), 544–554. doi:10.1016/j.cct.2013.09.015
132. Sherwood, N. E., JaKa, M. M., Crain, A. L., Martinson, B. C., Hayes, M. G., & Anderson, J. D. (2015) Pediatric Primary Care-Based Obesity Prevention for Parents of Preschool Children: A Pilot Study. *Childhood Obesity*, 11(6), 674-682. doi:10.1089/chi.2015.0009
133. Sherwood, N. E., Levy, R. L., Langer, S. L., Senso, M. M., Crain, A. L., Hayes, M. G., … Jeffery, R. W. (2013). Healthy homes/healthy kids: A randomized trial of a pediatric primary care-based obesity prevention intervention for at-risk 5–10year olds. *Contemporary Clinical Trials*, *36*(1), 228–243. doi:10.1016/j.cct.2013.06.017
134. Skouteris, H., Hill, B., McCabe, M., Swinburn, B., & Busija, L. (2015). A parent-based intervention to promote healthy eating and active behaviours in pre-school children: Evaluation of the MEND 2-4 randomized controlled trial. *Pediatric Obesity*, *11*(1), 4–10. doi:10.1111/ijpo.12011
135. Skouteris, H., McCabe, M., Swinburn, B., & Hill, B. (2010). Healthy eating and obesity prevention for preschoolers: A randomised controlled trial. *BMC Public Health*, *10*(1), 220. doi:10.1186/1471-2458-10-220
136. Slusser, W., Frankel, F., Robison, K., Fischer, H., Cumberland, W. G., & Neumann, C. (2012). Pediatric overweight prevention through a parent training program for 2–4 year old Latino children. *Childhood Obesity*, *8*(1), 52–59. doi:10.1089/chi.2011.0060
137. Smith, J. D., Montaño, Z., Dishion, T. J., Shaw, D. S., & Wilson, M. N. (2014). Preventing weight gain and obesity: Indirect effects of the family check-up in early childhood. *Prevention Science*, *16*(3), 408–419. doi:10.1007/s11121-014-0505-z
138. Sobko, T., Svensson, V., Ek, A., Ekstedt, M., Karlsson, H., Johansson, E., … Marcus, C. (2011). A randomised controlled trial for overweight and obese parents to prevent childhood obesity - early STOPP (STockholm obesity prevention program). *BMC Public Health*, *11*(1), 336. doi:10.1186/1471-2458-11-336
139. Spears-Lanoix, E. C., McKyer, E. L. J., Evans, A., McIntosh, W. A., Ory, M., Whittlesey, L., … Warren, J. L. (2015) Using Family-Focused Garden, Nutrition, and Physical Activity Programs To Reduce Childhood Obesity: The Texas! Go! Eat! Grow! Pilot Study. *Childhood Obesity*, 11(6), 707-714. doi:10.1089/chi.2015.0032
140. Spence, A. C., McNaughton, S. A., Lioret, S., Hesketh, K. D., Crawford, D. A., & Campbell, K. J. (2013). A health promotion intervention can affect diet quality in early childhood. *Journal of Nutrition*, *143*(10), 1672–1678. doi:10.3945/jn.113.177931
141. Tanvig, M., Vinter, C. A., Jørgensen, J. S., Wehberg, S., Ovesen, P. G., Beck-Nielsen, H., … Jensen, D. M. (2015). Effects of lifestyle intervention in pregnancy and Anthropometrics at birth on offspring metabolic profile at 2.8 years: Results from the lifestyle in pregnancy and offspring (LiPO) study. *The Journal of Clinical Endocrinology & Metabolism*, *100*(1), 175–183. doi:10.1210/jc.2014-2675
142. Taveras, E. M., Blackburn, K., Gillman, M. W., Haines, J., McDonald, J., Price, S., & Oken, E. (2010). First steps for Mommy and me: A pilot intervention to improve nutrition and physical activity behaviors of Postpartum mothers and their infants. *Maternal and Child Health Journal*, *15*(8), 1217–1227. doi:10.1007/s10995-010-0696-2
143. Taveras, E. M., McDonald, J., O’Brien, A., Haines, J., Sherry, B., Bottino, C. J., … Koziol, R. (2012). Healthy habits, happy homes: Methods and baseline data of a randomized controlled trial to improve household routines for obesity prevention. *Preventive Medicine*, *55*(5), 418–426. doi:10.1016/j.ypmed.2012.08.016
144. Taylor, B. J., Heath, A.-L. M., Galland, B. C., Gray, A. R., Lawrence, J. A., Sayers, R. M., … Taylor, R. W. (2011). Prevention of overweight in infancy (POI.nz) study: A randomised controlled trial of sleep, food and activity interventions for preventing overweight from birth. *BMC Public Health*, *11*(1), . doi:10.1186/1471-2458-11-942
145. Towey, M., Harrell, R., & Lee, B. (2011). Evaluation of “One body, One Life”: A community-based family intervention for the prevention of obesity in children. *Journal of Obesity*, *2011*, 1–7. doi:10.1155/2011/619643
146. Tucker, S. J., Ytterberg, K. L., Lenoch, L. M., Schmit, T. L., Mucha, D. I., Wooten, J. A., … Mongeon Wahlen, K. J. (2013). Reducing pediatric overweight: Nurse-delivered Motivational interviewing in primary care. *Journal of Pediatric Nursing*, *28*(6), 536–547. doi:10.1016/j.pedn.2013.02.031
147. Verbestel, V., De Coen, V., Van Winckel, M., Huybrechts, I., Maes, L., & De Bourdeaudhuij, I. (2013). Prevention of overweight in children younger than 2 years old: A pilot cluster-randomized controlled trial. *Public Health Nutrition*, *17*(06), 1384–1392. doi:10.1017/s1368980013001353
148. Walton, K., Filion, A. J., Gross, D., Morrongiello, B., Darlington, G., Simpson, J. R., … Haines, J. (2015) Parents and Tots Together: Pilot randomized controlled trial of a family-based obesity prevention intervention in Canada. *Canadian Journal of Public Health*, 106(8), e555-e562. doi:10.17269/CJPH.106.5224
149. Ward, D. S., Vaughn, A. E., Bangdiwala, S. I., Campbell, M., Jones, D. J., Panter, A. T., & Stevens, J. (2011). Integrating a family-focused approach into child obesity prevention: Rationale and design for the my parenting SOS study randomized control trial. *BMC Public Health*, *11*(1), . doi:10.1186/1471-2458-11-431
150. Weaver, S. P., Kelley, L., Griggs, J., Weems, S., & Meyer, M. R. U. (2014). Fit and healthy family cAMP for engaging families in a child obesity intervention: a community health center pilot project. *Family & community health*, *37*(1), 31-44.
151. Wen, L. M., Baur, L. A., Simpson, J. M., Rissel, C., Wardle, K., & Flood, V. M. (2012). Effectiveness of home based early intervention on children’s BMI at age 2: Randomised controlled trial. *BMJ*, *344*(jun26 3), e3732–e3732. doi:10.1136/bmj.e3732
152. Wen, L. M., Baur, L. A., Simpson, J. M., Xu, H., Hayes, A. J., Hardy, L. L., … Rissel, C. (2015). Sustainability of effects of an early childhood obesity prevention trial over time. *JAMA Pediatrics*, *169*(6), 543. doi:10.1001/jamapediatrics.2015.0258
153. Whaley, S. E., McGregor, S., Jiang, L., Gomez, J., Harrison, G., & Jenks, E. (2010). A WIC-Based intervention to prevent early childhood overweight. *Journal of Nutrition Education and Behavior*, *42*(3), S47–S51. doi:10.1016/j.jneb.2010.02.010
154. Willis, T. A., George, J., Hunt, C., Roberts, K. P. J., Evans, C. E. L., Brown, R. E., & Rudolf, M. C. J. (2013). Combating child obesity: Impact of HENRY on parenting and family lifestyle. *Pediatric Obesity*, *9*(5), 339–350. doi:10.1111/j.2047-6310.2013.00183.x
155. Yam, P. S., Morrison, R., Penpraze, V., Westgarth, C., Ward, D. S., Mutrie, N., … Reilly, J. J. (2012). Children, parents, and pets exercising together (CPET) randomised controlled trial: Study rationale, design, and methods. *BMC Public Health*, *12*(1), . doi:10.1186/1471-2458-12-208
156. Yilmaz, G., Demirli Caylan, N., & Karacan, C. D. (2014). An intervention to preschool children for reducing screen time: A randomized controlled trial. *Child: Care, Health and Development*, *41*(3), 443–449. doi:10.1111/cch.12133
157. Yin, Z., Parra-Medina, D., Cordova, A., He, M., Trummer, V., Sosa, E., … Ramirez, A. (2012). Míranos!Look at us, we are healthy! An environmental approach to early childhood obesity prevention. *Childhood Obesity*, *8*(5), 429–439. doi:10.1089/chi.2011.0125
158. Ziebarth, D., Healy-Haney, N., Gnadt, B., Cronin, L., Jones, B., Jensen, E., & Viscuso, M. (2012). A community-based family intervention program to improve obesity in Hispanic families. *WMJ*, *111*(6), 261-6.
159. Zoorob, R., Buchowski, M. S., Beech, B. M., Canedo, J. R., Chandrasekhar, R., Akohoue, S., & Hull, P. C. (2013). Healthy families study: Design of a childhood obesity prevention trial for Hispanic families. *Contemporary Clinical Trials*, *35*(2), 108–121. doi:10.1016/j.cct.2013.04.005
