# Supplementary material for: Family-based childhood obesity prevention interventions: a systematic review and quantitative content analysis
Source: Int J Behav Nutr Phys Act. 2017 Aug 24;14:113. doi: 10.1186/s12966-017-0571-2 (PMC5571569; doi:10.1186/s12966-017-0571-2)
Supplement: Supplementary file 3 — Intervention characteristics of family-based childhood obesity prevention interventions separating studies with evaluations from protocols. (DOCX 116 kb) [file 12966_2017_571_MOESM3_ESM.docx]

|  | **All Interventions (n=119)**  n (%) | **Interventions with evaluations (n=85)**  n (%) | **Interventions with protocols (n=34)**  n (%) |
| --- | --- | --- | --- |
| Geographic Region  United States  Europe/United Kingdom  Australia/New Zealand  Canada  Other^1^ | 66 (56)  30 (25)  10 (8)  6 (5)  7 (6) | 50 (59)  17 (20)  8 (9)  4 (5)  6 (7) | 16 (47)  13 (38)  2 (6)  2 (6)  1 (3) |
| Age of target child^2^  Prenatal  0-1 years  2-5 years  6-10 years  11-13 years  14-17 years | 10 (8)  29 (24)  51 (43)  42 (35)  25 (21)  8 (7) | 6 (7)  20 (24)  35 (41)  31 (36)  20 (24)  7 (8) | 4 (12)  9 (26)  16 (47)  11 (32)  5 (15)  1 (3) |
| Setting^2^ |  |  |  |
| Home | 33 (28) | 26 (31) | 7 (21) |
| Primary care/health clinic | 32 (27) | 21 (25) | 11 (32) |
| Community-based | 39 (33) | 29 (34) | 10 (29) |
| School | 21 (18) | 17 (20) | 4 (12) |
| Childcare/preschool  Multi-setting | 11 (9)  24 (20) | 7 (8)  18 (21) | 4 (12)  6 (18) |
| Not setting specific/Unclear | 11 (9) | 4 (5) | 7 (21) |
| Length of intervention |  |  |  |
| <13 weeks (<3 months) | 35 (29) | 28 (33) | 7 (21) |
| 13-51 week (3-11.9 months) | 47 (40) | 36 (42) | 11 (33) |
| 52 weeks or more (12 months or more) | 33 (28) | 18 (21) | 15 (44) |
| Unclear | 4 (3) | 3 (4) | 1 (3) |
| Delivery approach^2^  In-person delivery  Technology-based delivery | 101 (85)  27 (23) | 74 (87)  15 (18) | 27 (79)  12 (35) |
| Evaluation Design  Randomized-controlled trial design | 87 (73) | 55 (65) | 32 (94) |
| Recipients of intervention activities^2^  Children  Adults | 65 (55)  119 (100) | 53 (62)  85 (100) | 12 (35)  34 (100) |
| Behavioral domains targeted^2^ |  |  |  |
| Diet | 107 (90) | 76 (89) | 31 (91) |
| Physical activity | 97 (82) | 68 (80) | 29 (85) |
| Media use | 65 (55) | 49 (58) | 16 (47) |
| Sleep | 24 (20) | 14 (16) | 10 (29) |
| Funding source^2^  Federal  Foundation  Corporate  University  Unclear | 75 (63)  50 (42)  21 (18)  23 (19)  8 (7) | 47 (55)  39 (46)  15 (18)  17 (20)  7 (8) | 28 (82)  11 (32)  6 (18)  6 (18)  1 (3) |
| Theory^2^  Social Cognitive Theory  Parenting Styles  Ecological Framework  Transtheoretical Model of Behavior Change  Health Belief Model  Theory of Planned Behavior  Other  Unclear | 49 (41)  20 (17)  20 (17)  10 (8)  8 (7)  6 (5)  23 (19)  34 (29) | 33 (39)  11 (13)  13 (15)  7 (8)  6 (7)  2 (2)  18 (21)  31 (36) | 16 (47)  9 (26)  7 (21)  3 (9)  2 (6)  4 (12)  5 (15)  3 (9) |
| ^1^Other: Mexico/Central America- 2, South America- 2, Asia- 2, Middle East- 1; ^2^Groups are not mutually exclusive thus totals may exceed 100% | | | |
